# Metabolic status, reproductive, and productive performances of transition dairy cows as affected by dietary rumen-protected choline supplementation

**DOI:** 10.5455/javar.2024.k827

**Published:** 2024-09-30

**Authors:** Ratchataporn Lunsin, Damrongchai Sokantat, Taina Silvestre, Helio Rezende Lima Neto, Thong Jin Koh, Fei Sun, Chittraporn Yeanpet, Ruangyote Pilajun

**Affiliations:** 1 Program in Animal Science, Faculty of Agriculture, UbonRatchathaniRajabhat University, UbonRatchathani, Thailand; 2 Ruminant Technical Services, Animal Nutrition and Health, Asia Pacific, Kemin Industries, Inc., Des Moines, IA, USA; 3 Department of Animal Science, Faculty of Agriculture, UbonRatchathani University, UbonRatchathani, Thailand

**Keywords:** Rumen-protected choline, energy balance, milk production, transition dairy cows

## Abstract

Research articles about the effects of rumen-protected choline (RPC) supplementation on metabolic response, and reproductive and productive performances in transitional dairy cows were reviewed and presented. Analysis was conducted on 32 research papers that were published. The papers examined treatments without RPC supplementation and RPC supplementation levels varying from 10 to 100 gm/day. The feeding duration of RPC started from 40 to 140 days prepartum and continued until 20 to 140 days postpartum in multiparous dairy cows. Studies indicated that adding herbal choline (Cho) to the diet of transition dairy cows resulted in increased milk production and improved milk quality, leading to enhanced energy balance and reduced oxidative stress. The concentration and yield of IgG in colostrum provide passive immunity to dairy newborns and can be enhanced by dietary Cho supplementation. The inconsistent effects of RPC supplementation on reproduction may be due to several factors such as heat stress, genetics, and management. RPC supplementation improved the transition dairy cows’ milk yield and quality, but dosage response was not observed as in the prior publication. Remarkably, the length of RPC supplementation had a positive correlation with an increase in milk yield. Based on this review, 45–50 gm/day dietary RPC supplementation between 3 weeks pre-calving to 8 weeks post-calving is suggested to increase at least 10% milk yield in dairy cows.

## Introduction

**Figure 1.**
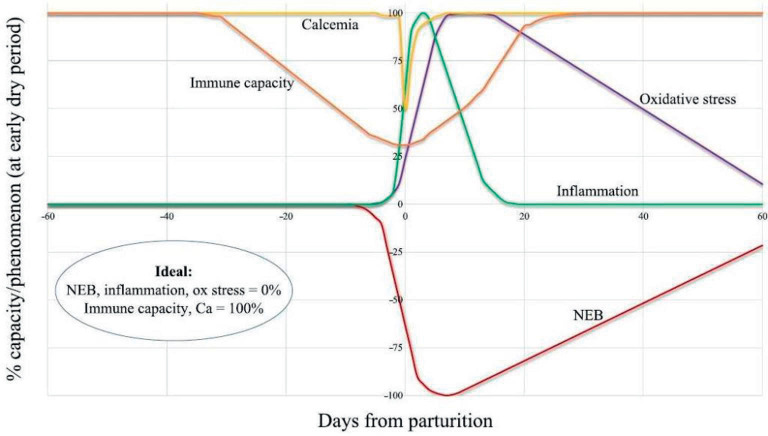
Theoretical pattern of changes in the main physiological aspects of healthy subjects during the transition period (Trevisi and Minuti, 2018; Cited by [9]).

The transition period, 3 weeks before to 3 weeks after calving, is one of the most critical physiological stages in dairy cattle. Numerous immunological and metabolic changes occur during this time, such as oxidative stress status, hypocalcemia, liver dysfunction, negative energy balance (NEB), and an overt systemic inflammatory response [1]. The transition cows face micronutrient deficits and NEB because of their decreased feed intake and increased energy and nutritional requirements for the synthesis of colostrum and milk. The NEB causes cows’ body fat to be mobilized into non-esterified fatty acids (NEFAs), which leads to an increase in blood levels of beta-hydroxybutyric acid (BHBA) [2]. If these systems remain out of balance for a long time, cows may be at a higher risk of disease, encounter negative reproductive results, and have decreased milk production and quality. While these modifications are a typical adaptive process in high-yielding cows, several metabolic and infectious illnesses develop when a cow is unable to adjust to this metabolic challenge, which impairs the productive and reproductive efficiency after the transition period. During the transition period, health issues can cause a physiological imbalance. This means that the animals’ regulatory mechanisms are not enough for them to function optimally, increasing the risk of various digestive, metabolic, and infectious problems (Figs. 1 and 3). To minimize health problems and maximize productivity and profitability for upcoming lactation, a smooth transition is crucial. It might be helpful to detect these diseases early to prevent further production losses. Although the transition dairy cow has been generally reviewed, the level, dose, and time duration of rumen-protected choline (RPC) supplementation to transition dairy cows are still not clear. Therefore, the present review article aims to find the suitable level and application of RPC in the transition period of dairy cows to improve production performances.

## Choline (Cho) Metabolism in Dairy Cows

**Figure 2.**
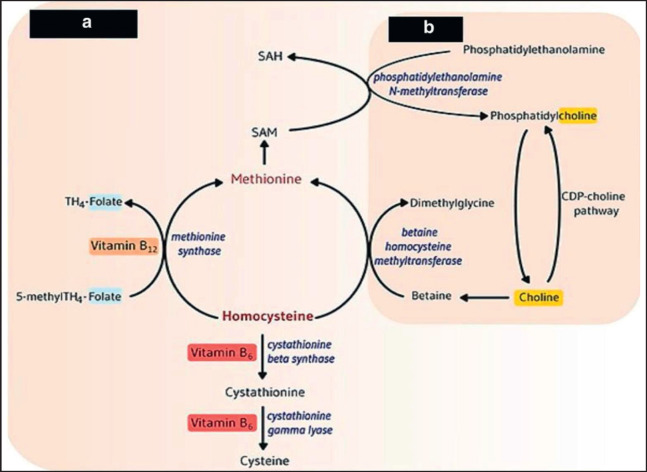
Methylation reaction pathway of homocysteine to form Met as related to Cho metabolism [3].

Methionine (Met) is seen as a critical amino acid (AA) that limits milk production. Both Met and Cho are crucial nutrients as they provide methyl groups in ruminants. The function of Met is to act as a supplier of methyl groups for the creation of Cho, and add Cho supports to reserve betaine for the production of sulfur-containing AAs (Met) to enhance animal performance (Fig. 2) [3]. Cho is an essential nutrient for many animals. It is often referred to as a vitamin but is not a co-factor in enzymatic reactions. Suppressed growth rates, renal dysfunction, and the onset of fatty liver are examples of deficiency symptoms. Cho is crucial for the normal function of all cells [4].

Phosphatidylcholine, a phospholipid found in all cell membranes and lipoproteins that carry lipids through the circulatory system, is the most prevalent kind of Cho in biological systems [5]. Cho is found in various forms in humans and animals. Acetylcholine, a crucial neurotransmitter for brain and neuromuscular function, betaine, an oxidative intermediate of Cho that provides a methyl group for the conversion of homocysteine to Met, and glycerophosphocholine, which functions as an organic osmolyte in cells like Bet, are among the water-soluble Cho metabolites [6]. Phosphatidylcholine, sphingomyelin, and lysophosphatidylcholine, which are lipid-soluble metabolites containing Cho, are structural elements found in mammalian membranes. Phosphatidylcholine plays a crucial role in the production of very low-density lipoprotein (VLDL), which is important for transporting triacylglycerol out of the liver.

## Effect of Herbal Cho Supplementation on Transition Dairy Cows

Antibiotic supplementation has been demonstrated to raise the risk of antibiotic residues, which may endanger animal and human health. Therefore, alternative and secure supplements for dairy animals were required. In comparison to other inorganic feed supplements or antibiotics, herbal feed supplementation has been demonstrated to be significantly safer for farm animals. It is anticipated that the application of herbal supplements during the transition period will lead to the necessary alterations to the body condition, reproduction, oxidative status, energy balance, and production of dairy animals. Through channelizing nutrient availability, maintaining proper body condition, and improving energy balance, the review examined the effects of supplementing herbal Cho (Indian native herbal plants) in dairy animals during the transition period and found far-reaching beneficial effects on their production, reproduction, and health performances [7]. Herbal mixtures with conjugates of Cho (Biocholine, BIO) and Met have increased milk yield in transition dairy cattle, as reported [8]. Supplementing cows with 15 gm/day of BIO increased the amount of fat-corrected milk but did not affect the chemical composition of the milk. Plasma glucose and aspartate transferase activity decreased with BIO supplementation. Herbal Cho (15 gm/day) and herbal Met (10 gm/day) supplements have been shown to enhance milk production while lowering the protein level, but not the lactose, fat, non-fatty solids, or total solids contents [10]. This is consistent with the positive effects of Cho addition from the feed plant additive at 0.071% of the diet, which decreased mastitis, abortions, and respiratory issues while increasing milk yield and fertility in primiparous cows and lowering the need for herd replacements [17]. In addition, crossbred cows supplemented with 20 gm herbal biocholine powder per day during the transition period, 3 weeks before to 3 weeks post-parturition, had increased lipid peroxidation, whereas, decreased superoxide dismutase and glutathione [18].

**Figure 3.**
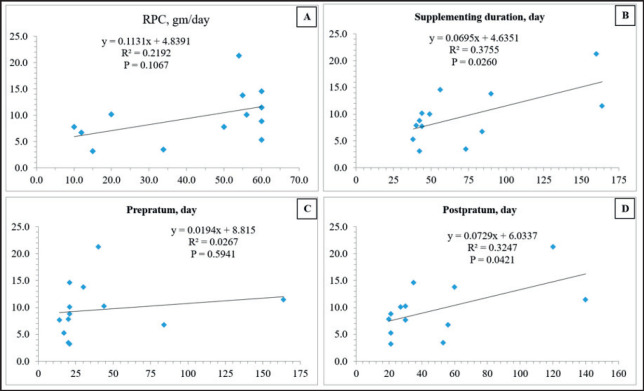
The linear regression between milk yield increased (%; *Y* axis) and dose (A, gm/day), total day (B), day prepartum (C), and until day postpartum (D; *X*-axis) of RPC supplementation in dairy cows.

**Table 1.** Effect of dietary RPC supplementation on reproductive performance of transition dairy cows.

Level and duration of supplementation	Source of RPC	Reproductive performance	Reference
60 gm/day RPC (17.3 gm/day of Cho chloride), 17 days pre- to 21 days postpartum	ReaShure, Balchem Corp., New Hampton, NY	- Pregnancy at first artificial insemination tended to be greater - The proportion of pregnant cows did not differ - The calf tended to have greater daily gain	Zenobi et al. [11]
10 gm/day RPC, 20 days pre- to 20 days postpartum	Herb-AllTM, Switzerland, Mels	- The first peak of P4 earlier - A shorter first insemination time - Decreased insemination rate - A shorter period between calvings	Mecionyte et al. [12]
54 gm/day RPC, 40 days pre- to 120 days postpartum	CholiPEARL^TM^, Kemin Industries, Des Moines IA	- Days open decreased from 164.2 to 131.3 - Conception rate (%) was increased from 55.6% to 77.8%	Amrutkar et al. [13]
55 gm/day RPC, 30 days pre- to 60 days postpartum	Kemin Industries, Des Moines, IA	- Estrous cyclicity, services to conception ratio, conception & pregnancy rates were similar - Decreased service period (days)	Acharya et al. [14]
15 gm/day RPC, 25 days pre- to 80 days postpartum	ReaShure, Balchem Corp., New Hampton, NY	Ovarian cyclicity or pregnancies per insemination did not differ	Lima et al. [15]
15 gm/day RPC, 4 weeks pre- to 20 weeks postpartum	COL 24^®^ (Kemin Industries, Des Moines, IA)	- Days open and services per conception were decreased when RPC was supplemented with rumen-protected Met - Incidence of health problems such as retained placenta, uterine problem, and dystocia were decreased	Ardalan et al. [16]

## Effect of Rumen-Protected Cholineon Colostrum Yields and Quality

Colostrum is an essential nutrient source that gives dairy newborn calves passive immunity, through the absorption of immunoglobins across the gut. Cho is a bioactive micronutrient that serves as a building block for numerous other compounds, and several studies supplemented the RPC to support performance in postpartum dairy cows. Therefore, dietary supplementation of RPC may affect colostrum yields and quality in periparturient dairy cows. The amount of colostrum generated by transition Holstein cows supplemented with 45 gm/day (20.4 gm/day of Cho ions) of RPC was reported to rise without influencing the concentration of antibodies [19]. Furthermore, dietary Cho supplementation enhanced phosphocholine and trimethylamine N-oxide concentrations in colostrum. This is following findings that cows given 60 gm per day of RPC before delivery had increased IgG production and concentration in their colostrum [11]. Changing the levels of Cho metabolites in colostrum resulting from RPC supplementation could have an impact on the health and productivity of dairy cows going through parturition as well as newborn calves. However, 60 gm/day RPC did not affect the yield or the composition and IgG of colostrum, although somatic cell count was decreased when supplemented during 21 days pre- to 21 days post-partum [20]. The mammary gland secretes colostrum, but its main function is to move vast quantities of plasma IgG to epithelial cells over the mammary barrier [21]. However, it is uncertain how Cho or phospholipids produced from it affect the amount of IgG in colostrum. Proliferation of mammary cells [22] and improved Ig transfer from plasma to the mammary epithelial cell [21] are potential research areas.

## Effect of RPC on Reproductive Performance of Transition Dairy Cows

Lipid metabolism plays a key role in the transition period of dairy cows and therefore is also critical to reproduction. There have been reports of reduced pregnancy rates and delayed cyclicity in cows with elevated blood ketone levels following parturition; whereas, feeding RPC positively impacts reproduction. However, varying effects of RPC supplementation on reproduction have been reported in Table 1. While some studies [12,13] supported the idea that transition dairy cows receiving RPC performed better reproductively, other studies [11,14,15] found no difference in the effects of RPC or even adverse consequences. This is in line with a study that collected data from multiple studies through meta-analysis and discovered varying degrees of benefit from RPC supplementation on dairy cow performance [23]. In addition, according to a study [16], transition dairy cows’ reproductive performance may be impacted by supplementing with rumen- or unprotected Met, which could lead to better oocyte quality, fewer days open, and fewer services per conception. Other factors, not only nutrition, also impact the reproductive characteristics of dairy cattle during the transition period. Heat stress plays an important role in the pregnancy rate of dairy cows, especially in tropical climates [37], while genetics [38], disease, health and herd management, and reproductive services also have significant impacts and concerns [39].

**Table 2.** Effect of dietary RPC supplementation on milk production of transition dairy cow.

Level of supplement	Duration of supplement	Milk yield, % change	Milk composition	Reference
60 gm/day RPC (17.3 gm/day of Cho chloride)	17 days pre- to 21 days postpartum	Tended + 5.33%	ns	Zenobi et al. [11]
10 gm/day RPC	20 days pre- to 20 days postpartum	+ 7.87%	+ % fat - SCC	Mecionyte et al. [12]
54 gm/day RPC	40 days pre- to 120 days postpartum	+ 21.3%	+ %fat & protein	Amrutkar et al. [13]
<33.8 gm/day of Cho chloride	20 days pre- to 53 days postpartum	+ 3.46%	+ fat & protein yield	Humer et al. [23]
60 gm/day RPC with/without 18 gm/day RPM	4 weeks pre- to 20 weeks postpartum	+ 11.5%	ns	Ardalan et al. [16]
60 gm/day RPC (12.9 gm/day of choline ion)	21 days pre- to 21 days postpartum	+ 8.84% FCM	+ fat yield	Bollatti et al. [24]
15 gm/day RPC	21 days pre- to 21 days postpartum	+ 3.17% FCM	+ % fat	Sun et al. [25]
50 gm RPC plus 1,000 IU vitamin E	14 days pre- to 30 days postpartum	Tendency + 7.77%	ns	Pinotti et al. [26]
12.9 gm/day of choline ion	Transition period	+ 4.82	+ fat & protein yield	Arshad et al. [27]
60 gm/day RPC (13.0 gm/day of choline ion)	3 weeks pre- to 5 weeks postpartum	+ 14.6% in primiparous cows	ns	Potts et al. [28]
6-12 gm/day RPC with low RUP	28 days pre- to 56 days postpartum	+ 6.74%	- % protein	Hartwell et al. [29]
60 gm/day RPC (25% choline Cl)	21 days pre- to 21 days postpartum	ns	ns	Guretzky et al. [30]
55 gm/day RPC	30 days pre- to 60 days postpartum	+ 13.8%	+ % fat & protein	Acharya et al. [31]
50 and 100 gm/day RPC (18.8% choline Cl)	21 days pre- to 45 days postpartum	ns	+ % protein & total solids	Leiva et al. [32]
100 and 200 gm/day RPC	21 days pre- to 60 days postpartum	+ 11.8%	+ % protein	Elek et al. [33]
56 gm/day RPC	3 weeks pre- to 28 days postpartum	+ 3.90-16.3%	ns	Zahra et al. [34]
45, 60, 75 gm/day RPC	21 days pre- to 63 days postpartum	ns	ns	Piepenbrink and Overton [35]
20 gm/day RPC	14 days pre- to 30 days postpartum	+ 10.2%	ns	Pinotti et al. [36]

ns, non-significant differ.

## Effect of RPC on the Inflammatory and Metabolic Status and Health of Transition Dairy Cows

Due to differences in the level and duration of supplementation, and the capability of protecting Cho from rumen degradation of each product, the response of the inflammatory, metabolic status, and health in transition dairy cows to RPC supplementation has been varying. However, the favorable reaction might be connected to the beneficial impact of Cho on hepatic gene networks, which helps in the production and release of VLDL. The study found that giving 12.9 gm/day of RPC ion to parous Holstein cows from 21 days before 21 days postpartum increased the amount of triacylglycerol in the liver and the concentration of BHBA in plasma during the first 21 days postpartum. However, the levels of inflammatory markers and liposoluble vitamins in plasma remained unchanged [24]. Agree with previous studies [25,26,40] that reported dietary supplementation with RPC improved NEB by enhanced fatty acid processing and VLDL synthesis, decreased the plasma concentrations of NEFAs, BHBA, total cholesterol, and low-density lipoprotein cholesterol. In addition, phosphatidylcholine, triglycerides, VLDL, vitamin E, and Met concentrations in blood plasma were higher in Karan-Fries cows given 54 gm of RPC 40 days before and 120 days following calving than in the control group [13]. However, no effects of RPC on blood metabolites, liver triacylglycerol contents, or energy balance have also been reported [11,23].

Improving immune status and oxidative stress during the transition period of dairy cows by RPC supplementation has been reported in several studies [14,25,41,42]. It was found that total immunoglobulin was increased while serum levels of total antioxidant capacity and thiobarbituric acid reactive substances were reduced [43], monocyte phagocytosis capacity was increased [42], and greater phagocytosis and oxidative burst capabilities upon pathogen challenge [44] significantly in cows fed RPC during the transition period. By lowering the frequency of ketosis and mastitis, feeding RPC to dairy cows during late gestation and the first 3 months of lactation enhanced health [15]. Nevertheless, RPC feeding to primiparous cows before calving had a varied effect on their health. The frequency of metritis increased, whereas the incidence of retained fetal membranes and mastitis infections per cow declined. However, based on the results of several papers that referred to it, it was difficult to conclude or suggest a suitable level of RPC supplementation for a suitable metabolic status of transition dairy cows. It may be due to the proper level of NEFA, BHBA, or other metabolites in the blood, which could be different in each animal status, such as prepartum and postpartum, low milk yield, and so on.

## Effect of RPC on Productive Performance of Transition Dairy Cows

Milk yield and composition are also important traits of dairy production. Supplementation of RPC can increase both quantity and quality traits, especially fat concentration (Table 2) mostly due to improving daily feed intake and energy metabolism. The results of this review corroborate those of a prior study [27] that employed meta-analytic techniques to assess the impact of supplemental RPC on dairy cow productivity and discovered that supplementation RPC raised milk, energy-corrected milk, fat, and protein yields. The result from the meta-analysis and review papers, not only increased dry matter intake and milk yield when cows received RPC but also increased milk fat yield and milk protein yield [3,23,45]. However, responses to RPC during the periparturient period may rely upon the availability of Met, according to several trials’ differing findings [28]. In accordance with a study [29], that discovered no changes were made to milk production, content, or consumption when 6-12 gm/day of RPC was fed during the prepartum phase and for the full 120 days of lactation. In addition, due to the only 2.3 kg increase in FCM yield observed for cows supplemented with 60 gm/day, RPC was not statistically significant. Benefits from RPC supplementation seem most likely for cows that are more susceptible to fatty liver and for diets that have a low passage of Met to the small intestine [30]. Cho supplementation may be more effective when fed with basal diets that limit post-ruminal Met supply.

We found linear regression between increasing cow’s milk yield (%) and total day of supplementation (B) and the day before calving (C) (*p *< 0.05) but not dose (A) or day after calving (D) of RPC supplementation (Fig. 3). This is in accordance with other studies [2,23] that reviewed research articles and revealed that there were no dose-dependent effects of RPC supplementation on the milk yield of cows; nevertheless, the suggested doses for the transition period remain unknown. However, it indicated that RPC could supplement a long time before calving or long-term enough during the transition period. When compared among research, longer supplementation of RPC after calving showed more increase in milk yield [13,16,31,33]. However, the absence of the effect of long-term RPC supplementation on milk yield in some studies [35] may be due to the bioavailability of Cho from RPC to the small intestine of dairy cows.

## Conclusion

During the transition phase, RPC-supplemented animals change the plasma NEFA concentration and hepatic fat export, which may lower the risk of metabolic issues and enhance the milk yield and milk composition of transition dairy cows. Inconsistent effects of RPC supplementation on reproduction were illustrated while increasing both milk yield and quality was not responded with the amount of RPC. Nevertheless, increasing milk yield had a positive correlation with the length of RPC supplementation both from pre- to post-calving and after parturition. Based on the linear equations in Figure 3, 45–50 gm/day of RPC has to supplement in transition dairy cows’ ration at least 78 consecutive days from 3 week pre- to 8 week post-calving to increase 10% milk yield. Further research is required to apply diverse types of RPC to the farm level, obtainability of Cho from RPC to the small intestine may be suitable for different farm conditions. Cho supplementation improves hepatic lipid metabolism in many mammals; however, rumen microorganisms extensively degrade the majority of Cho present in feed ingredients and supplements [46]. Therefore, the bioavailability of Cho delivered by RPC products needs to be carefully evaluated in terms of its effectiveness in intestinal absorption.

## Data Availability

This paper is a review that draws from published articles that are cited and included in the manuscript’s reference lists.

## List of Abbreviations

AA, Amino acid; BHBA, Beta-hydroxybutyric acid; Cho, Choline; Met, Methionine; NEB, Negative energy balance; NEFA, Non-esterified fatty acids; RPC, Rumen-protected choline; VLDL, Very low-density lipoprotein.
